# Screening for fecal presence of colistin-resistant *Escherichia coli* and *mcr*-*1* and *mcr*-*2* genes in camel-calves in southern Tunisia

**DOI:** 10.1186/s13028-018-0389-1

**Published:** 2018-06-05

**Authors:** Mohamed Rhouma, Salma Bessalah, Imed Salhi, William Thériault, John Morris Fairbrother, Philippe Fravalo

**Affiliations:** 1 Department of Pathology and Microbiology, Faculty of Veterinary Medicine, University of Montreal, 3200 Sicotte St, Saint-Hyacinthe, QC J2S 2M2 Canada; 2 Livestock and Wildlife Laboratory, Arid Lands Institute (I.R.A), University of Gabès, Médenine, Tunisia

**Keywords:** Camel, Colistin resistance, *E. coli*, Human, MCR

## Abstract

Camels (*Camelus dromedarius*) are known to harbor multidrug resistant Gram-negative bacteria and to be involved in the transmission of various microorganisms to humans. Data on the occurrence of colistin resistant *Escherichia coli* as well as mobilized colistin resistance (*mcr*) genes in camels are lacking. We investigated the presence of colistin resistance and *mcr* (*1*–*2*) genes in *E. coli* from the feces of camels in Tunisia. Presumptive *E. coli* isolates from camel-calves in southern Tunisia were qualitatively screened for growth on Mueller–Hinton agar supplemented with 2 mg/L of colistin. The minimal inhibitory concentration of colistin was determined for isolates growing on this medium. All isolates were screened for the presence of the *mcr*-*1* and *mcr*-*2* genes by polymerase chain reaction without detecting any of these genes. However, one isolate was confirmed resistant to colistin and further testing of this isolate revealed it to be *Enterobacter cloacae*. Our study demonstrated absence of colistin resistance and of the *mcr*-*1* and *mcr*-*2* genes in *E. coli* isolated from camel feces in southern Tunisia. Thus, there is no evidence that camels represent a major source of *mcr* genes contamination for the local population or for tourists visiting southern Tunisia.

## Findings

One-humped camels (*Camelus dromedarius*) are raised extensively in southern Tunisia for the production of meat and milk as well as for activities related to Sahara tourism. One-humped camels are considered part of the cultural heritage and represents a national wealth for many countries [1]. Camel-calves have a high susceptibility to bacterial infections, particularly those caused by *Escherichia coli* [1–3]. In fact, colibacillosis in young camelids results in considerable economic losses in camel farms, being associated with mortality, growth retardation and medical treatment costs [1, 4]. The absence of antimicrobials approved to treat bacterial infections in dromedaries poses a major challenge for veterinarians working in this animal production [5]. The use of antimicrobials approved for ruminants, horses or other animal species in the treatment of sick camels has not been associated with satisfactory results because of the physiological particularities of this animal species [5]. Colistin sulfate (CS), a polypeptide antibiotic, is used off-label in Tunisia for the oral treatment of *Enterobacteriaceae* infections in camel-calves at the dose of 25,000 IU/kg twice a day for 3 consecutive days. Following the recent identification of a stable plasmid mediated mobilized colistin resistance (*mcr*-*1*) gene encoding for resistance to colistin in *Enterobacteriaceae,* this “novel” mechanism of colistin resistance has been widely studied in bacteria isolated from several origins such as farm animals, humans, food and the environment [6, 7]. In July and August 2017, we searched PubMed with the terms “colistin in dromedary or colistin in camels”, “colistin resistance in dromedary or in camels”, “MCR-1 in dromedary or in camels” and “*mcr* genes in dromedary or in camels”. However, no studies on fecal presence of plasmid-mediated colistin resistance genes (*mcr*-*1* to -*5*) or *E. coli* colistin resistance in camels were found. On the other hand, it was been recently reported that camels are a potential source of human contamination with multidrug resistant (MDR) bacteria such as extended-spectrum beta-lactamase (ESBL) producing *E. coli* or *Pseudomonas aeruginosa* [5, 8]. Moreover, co-localisation of *mcr*-*1* and ESBL genes on a unique plasmid has been reported [9]. The present study aimed (1) to determine the fecal prevalence of *E. coli* colistin resistance in camel-calves with and without diarrhea in Tunisia, and (2) to determine the prevalence of *mcr*-*1* and *mcr*-*2* genes among both colistin resistant and susceptible *E. coli* isolates.

**Fig. 1:**
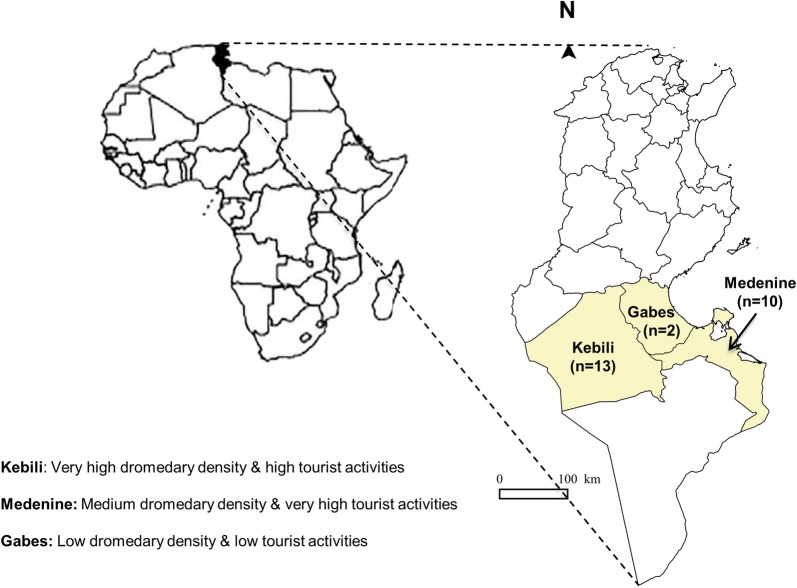
Geographic distribution of the sampled farms by district in southern Tunisia with the dromedary density and its involvement in tourist activities

In a previous study, the distribution of virulence genes, pathotypes, serogroups, phylogenetic groups, and antimicrobial resistance of presumptive *E. coli* isolated from camel-calves with and without diarrhea in Tunisia was investigated [2]. Fecal samples had been collected between January 2011 and April 2013 from 25 extensive camel farms located in three districts in southern Tunisia: Kebili (n = 13), Gabes (n = 2), and Medenine (n = 10) (Fig. 1) with an average flock size of 75 animals. Rectal swabs from 120 camel-calves aged between 1 and 3 months, with or without diarrhea, were suspended in buffered peptone water solution. Selected dilutions (10^−1^, 10^−2^, and 10^−3^) were plated on MacConkey agar. The plates were incubated aerobically for 24 h at 37 °C. Colonies growing on MacConkey agar with a morphology typical for *E. coli* (red or pink, non-mucoid colonies) were tested by polymerase chain reaction (PCR) for the β-glucuronidase (*uidA*) housekeeping gene, and PCR-positive colonies obtained (n = 52) were considered as presumptive *E. coli* isolates (Table 1) [10]. These isolates were stored at − 80 °C until further analysis.

**Table 1:** Distribution of possibly colistin resistant isolates according to their origin; camel- calves with or without diarrhea

Health status of camel-calves	Number of isolates	Species (number)	Colistin resistant isolates (number)	MIC of colistin value (mg/L)
No diarrhea	23	*E. coli* (n = 23)	0	–
Diarrhea	29	*E. coli* (n = 28) *E. cloacae* (n = 1)	0 *E. cloacae* (n = 1)	– 4

*Escherichia coli, Enterobacter cloacae*

After thawing, the 52 isolates were inoculated on Mueller–Hinton agar supplemented with CS at the concentration of 2 mg/L. Isolates demonstrating growth were considered as possibly colistin-resistant and were examined for minimum inhibitory concentration (MIC) of colistin, using the broth microdilution method in technical duplicate according to the guidelines of the Clinical Laboratory Standards Institute (CLSI) [11]. *Enterobacteriaceae* with colistin MICs > 2 mg/L were defined as resistant (R), and those with colistin MICs ≤ 2 mg/L as susceptible (S) according to the European Committee on Antimicrobial Susceptibility Testing (EUCAST) guidelines (http://www.eucast.org/clinical_breakpoints/). Isolates with colistin MICs > 2 mg/L were further identified using the API 20E system (bioMérieux, Quebec City, Canada).

Bacterial DNA extraction was performed using the Chelex^®^ method (Bio-Rad, ON, Canada). All isolates were screened by PCR for the presence of *mcr*-*1* and *mcr*-*2* genes, using primers and conditions as previously described [12, 13]. DNA from *E. coli* strains harbouring either *mcr*-*1* gene [14] or *mcr*-*2* gene [13] were used as positive control.

**Fig. 2:**
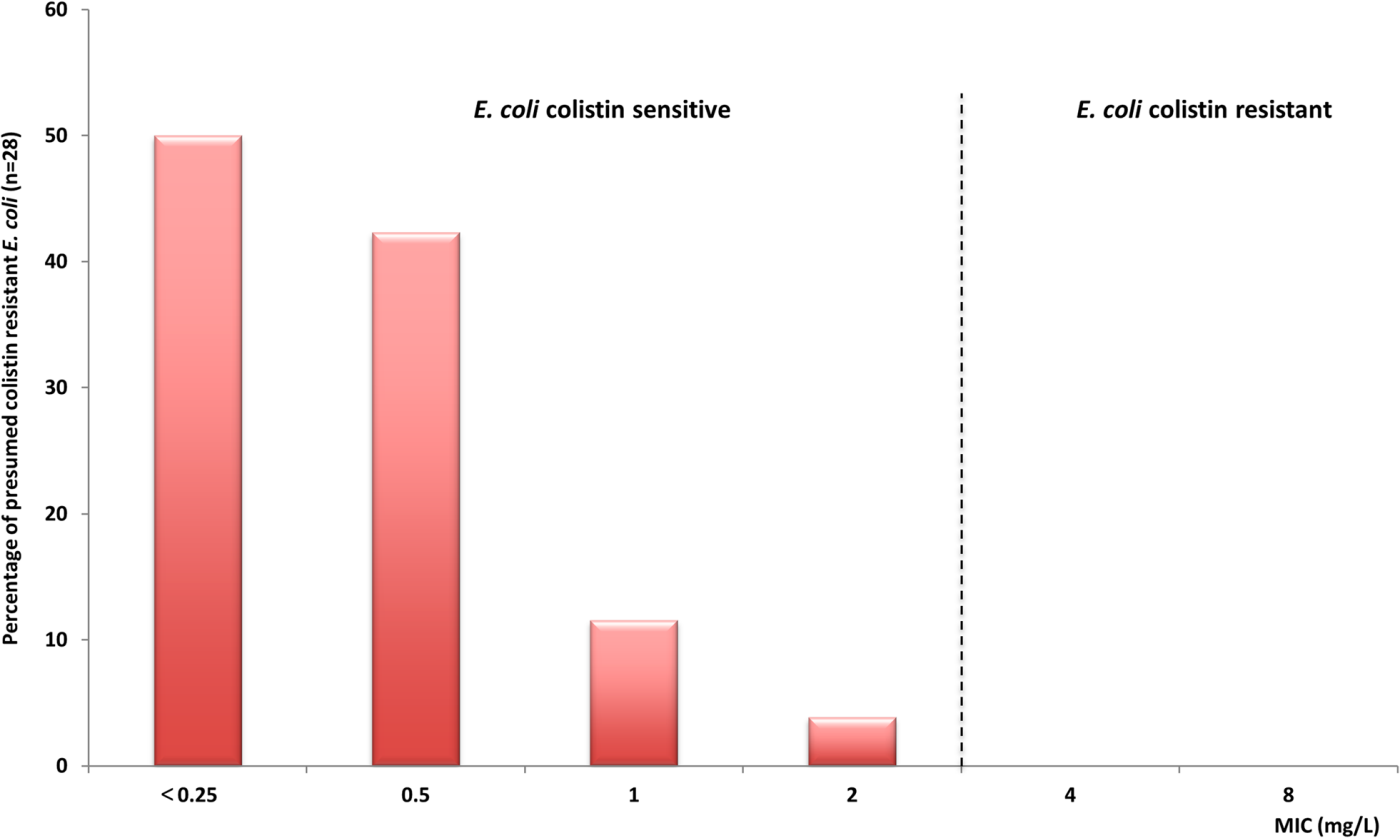
Distribution of minimum inhibitory concentrations (MICs) in *E. coli* isolates possibly resistant to colistin (n = 28) recovered from camel-calves with and without diarrhea

More than half of the presumptive *E. coli* isolates (55%) (n = 28) in both groups of animals were identified as possible colistin resistant (Fig. 2). However, only one of these isolates was confirmed to be resistant to colistin with an MIC value of 4 mg/L (Table 1). This resistant isolate, recovered from a camel-calf with diarrhea, was identified as *Enterobacter cloacae*.

Neither the *mcr*-*1* nor *mcr*-*2* gene were detected in any of the colistin resistant or susceptible isolates.

It is important to monitor colistin resistance in dromedary herds in Tunisia as the *mcr*-*1* gene is highly prevalent in food-producing animals (such as chickens) in this country [15]. In addition, Tunisia being an important tourist destination [16], close contact between camels and tourists visiting southern Tunisia may result in spread of resistant bacteria internationally.

Our finding that colistin-resistance and the *mcr* genes were absent in *E. coli* isolated from camel-calves in southern Tunisia suggest that dromedaries are not a major source of contamination with these genes for inhabitants as well as for tourists visiting this region. It should be noted that colistin-susceptible isolates were included in the current study, as previous studies have reported the identification of *mcr*-*1* gene among colistin-resistant and colistin-susceptible isolates [6, 17].

We report here for the first time the isolation of *E. cloacae* resistant to colistin from a diarrheic camel. However, this isolate did not harbor *mcr*-*1* or *mcr*-*2* genes. Similarly, colistin-resistant *E. cloacae* isolates with or without the *mcr*-*1* gene have been found in healthy people or primary care patients [18–20]. Currently, the mechanism of colistin resistance in *E. cloacae* strains in the absence of plasmid-borne colistin resistance genes (*mcr*-*1* to -*5*) remains poorly understood. Zhong et al. [21] recently identified 17 genes in the chromosome and one gene in pASM1 encoding efflux pump proteins in a highly colistin resistant *E. cloacae* strain. Nevertheless, mechanisms of colistin resistance not associated with plasmids in *Enterobacter* spp. require further investigation.

It should be emphasized that we had no information regarding the medical treatment history of the sampled camel-calves, specifically, whether or not colistin has been used in these dromedary farms. Additional studies are needed in order to assess the spread of colistin resistant bacteria and *mcr* genes among enteric bacteria of camel origin worldwide. Future surveys should take into consideration other areas where dromedary density is very high, such as the Arabian Peninsula where the *mcr*-*1* gene has been identified in *E. coli* isolates [22].

In the present study, the number of isolates obtained was limited because of the difficulty of handling camel-calves in extensive farms. However, we believe that our sampling was representative of dromedary production in Tunisia, as fecal samples were obtained from districts containing the highest density of dromedary farms.

As expected, the use of Mueller–Hinton agar supplemented with 2 mg/L of CS overestimated the frequency of presumed colistin resistant bacteria. But the culturing on this medium was only used as a screening method for reducing the number of isolates to be tested for colistin MIC. In addition, because of the poor diffusion of colistin in agar, it was always recommended to confirm presumed colistin resistant isolates, growing on colistin containing agar, by colistin CMI determination [23, 24].

The results suggest that camel-calves do not represent a major source of *mcr* gene contamination for the local population or for tourists visiting southern Tunisia. Nevertheless, it highlights the need to widen the scope of monitoring colistin resistance to other *Enterobacteriaceae* species than those conventionally identified in veterinary diagnostic laboratories, such as *E. cloacae*. Moreover, with the global initiatives for the establishment of a One Health antimicrobial resistance surveillance approach, camels should be also considered as an important animal species in the tracking of resistant bacteria at the human, animal and environment interface.

## Abbreviations

CLSI: Clinical and Laboratory Standards Institute
CS: colistin sulfate
ESBL: extended-spectrum beta-lactamase
EUCAST: European Committee on Antimicrobial Susceptibility Testing
DNA: deoxyribonucleic acid
MDR: multidrug resistant
MICs: minimal inhibitory concentrations
PCR: polymerase chain reaction

## References

[1] Al-Ruwaili MA, Khalil OM, Selim SA (2012). Viral and bacterial infections associated with camel (*Camelus dromedarius*) calf diarrhea in North Province, Saudi Arabia. Saudi J Biol Sci..

[2] Bessalah S, Fairbrother JM, Salhi I, Vanier G, Khorchani T, Seddik MM (2016). Antimicrobial resistance and molecular characterization of virulence genes, phylogenetic groups of *Escherichia coli* isolated from diarrheic and healthy camel-calves in Tunisia. Comp Immunol Microbiol Infect Dis.

[3] El Wathig M, Faye B (2016). Camel calf diarrhoea in Riyadh region, Saudi Arabia. J Camel Pract Res..

[4] Wernery U, Kinne J, Schuster RK, editors. Camelid infectious disorders. Colibacillosis. 2014, World Organisation for Animal Health (OIE). Paris, France; 2014. P. 100–5.

[5] Fadlelmula A, Al-Hamam NA, Al-Dughaym AM (2016). A potential camel reservoir for extended-spectrum beta-lactamase-producing *Escherichia coli* causing human infection in Saudi Arabia. Trop Anim Health Prod.

[6] Rhouma M, Beaudry F, Theriault W, Letellier A (2016). Colistin in pig production: chemistry, mechanism of antibacterial action, microbial resistance emergence, and one health perspectives. Front Microbiol..

[7] Rhouma M, Beaudry F, Letellier A (2016). Resistance to colistin: what is the fate for this antibiotic in pig production?. Int J Antimicrob Agents.

[8] Elhariri M, Hamza D, Elhelw R, Dorgham SM (2017). Extended-spectrum beta-lactamase-producing *Pseudomonas aeruginosa* in camel in Egypt: potential human hazard. Ann Clin Microbiol Antimicrob..

[9] Rhouma M, Letellier A (2017). Extended-spectrum beta-lactamases, carbapenemases and the *mcr*-*1* gene: is there a historical link?. Int J Antimicrob Agents.

[10] Walk ST, Alm EW, Gordon DM, Ram JL, Toranzos GA, Tiedje JM (2009). Cryptic lineages of the genus *Escherichia*. Appl Environ Microbiol.

[11] Rhouma M, Beaudry F, Thériault W, Bergeron N, Laurent-Lewandowski S, Fairbrother JM (2015). Gastric stability and oral bioavailability of colistin sulfate in pigs challenged or not with *Escherichia coli* O149: F4 (K88). Res Vet Sci.

[12] Liu YY, Wang Y, Walsh TR, Yi LX, Zhang R, Spencer J (2016). Emergence of plasmid-mediated colistin resistance mechanism MCR-1 in animals and human beings in China: a microbiological and molecular biological study. Lancet Infect Dis..

[13] Xavier BB, Lammens C, Ruhal R, Kumar-Singh S, Butaye P, Goossens H (2016). Identification of a novel plasmid-mediated colistin-resistance gene, *mcr*-*2*, in *Escherichia coli*, Belgium, June 2016. Euro Surveill.

[14] Perrin-Guyomard A, Bruneau M, Houée P, Deleurme K, Legrandois P, Poirier C (2016). Prevalence of mcr-1 in commensal *Escherichia coli* from French livestock, 2007 to 2014. Euro Surveill.

[15] Grami R, Mansour W, Mehri W, Bouallègue O, Boujaâfar N, Madec J (2016). Impact of food animal trade on the spread of *mcr*-*1*-mediated colistin resistance, Tunisia, July 2015. Euro Surveill.

[16] Halioui S, Schmidt M. Participatory decision-making for sustainable tourism development in Tunisia. In: Tourism, culture and heritage in a smart economy: Katsoni V, Upadhya A, Stratigea A, Editors. Third international conference IACuDiT, Athens. Springer proceedings in Business and economics; 2017. P. 323–38.

[17] Fernandes M, Moura Q, Sartori L, Silva K, Cunha M, Esposito F (2016). Silent dissemination of colistin-resistant *Escherichia coli* in South America could contribute to the global spread of the *mcr*-*1* gene. Euro Surveill.

[18] Baron S, Bardet L, Dubourg G, Fichaux M, Rolain JM (2017). MCR-1 plasmid-mediated colistin resistance gene detection in an *Enterobacter cloacae* clinical isolate in France. J Glob Antimicrob Resist..

[19] Zurfluh K, Stephan R, Widmer A, Poirel L, Nordmann P, Nuesch HJ (2017). Screening for fecal carriage of MCR-producing *Enterobacteriaceae* in healthy humans and primary care patients. Antimicrob Resist Infect Control..

[20] Lin J, Zhao F, Feng Y, Zong Z (2017). Draft genome sequence of a high-level colistin-resistant clinical strain of the *Enterobacter cloacae* complex. Genome Announc.

[21] Zhong C, Zhang C, Fu J, Chen W, Jiang T, Cao G (2018). Complete genome sequence of *Enterobacter cloacae* R11 reveals multiple genes potentially associated with high-level polymyxin E resistance. Can J Microbiol.

[22] Sonnevend A, Ghazawi A, Alqahtani M, Shibl A, Jamal W, Hashmey R (2016). Plasmid-mediated colistin resistance in *Escherichia coli* from the Arabian Peninsula. Int J Infect Dis..

[23] Rhouma M, Beaudry F, Theriault W, Bergeron N, Beauchamp G, Laurent-Lewandowski S (2016). *In vivo* therapeutic efficacy and pharmacokinetics of colistin sulfate in an experimental model of enterotoxigenic *Escherichia coli* infection in weaned pigs. Vet Res.

[24] Turlej-Rogacka A, Xavier BB, Janssens L, Lammens C, Zarkotou O, Pournaras S (2018). Evaluation of colistin stability in agar and comparison of four methods for MIC testing of colistin. Eur J Clin Microbiol Infect Dis.

